# Effect of the economic recession on pharmaceutical policy and medicine sales in eight European countries

**DOI:** 10.2471/BLT.13.129114

**Published:** 2014-06-16

**Authors:** Christine Leopold, Aukje K Mantel-Teeuwisse, Sabine Vogler, Silvia Valkova, Kees de Joncheere, Hubert GM Leufkens, Anita K Wagner, Dennis Ross-Degnan, Richard Laing

**Affiliations:** a World Health Organization (WHO) Collaborating Centre for Pharmaceutical Pricing and Reimbursement Policies, Gesundheit Österreich GmbH, Stubenring 6, 1010, Vienna, Austria.; b WHO Collaborating Centre for Pharmaceutical Policy and Regulation, Utrecht Institute for Pharmaceutical Sciences, Utrecht, Netherlands.; c IMS Institute for Healthcare Informatics, Philadelphia, United States of America (USA).; d Department of Essential Medicines and Pharmaceutical Policies, World Health Organization, Geneva, Switzerland.; e Department of Population Medicine, Harvard Medical School, Boston, USA.

## Abstract

**Objective:**

To identify pharmaceutical policy changes during the economic recession in eight European countries and to determine whether policy measures resulted in lower sales of, and less expenditure on, pharmaceuticals.

**Methods:**

Information on pharmaceutical policy changes between 2008 and 2011 in eight European countries was obtained from publications and pharmaceutical policy databases. Data on the volume and value of the quarterly sales of products between 2006 and 2011 in the 10 highest-selling therapeutic classes in each country were obtained from a pharmaceutical market research database. We compared these indicators in economically stable countries; Austria, Estonia and Finland, to those in economically less stable countries, Greece, Ireland, Portugal, Slovakia and Spain.

**Findings:**

Economically stable countries implemented two to seven policy changes each, whereas less stable countries implemented 10 to 22 each. Of the 88 policy changes identified, 33 occurred in 2010 and 40 in 2011. They involved changing out-of-pocket payments for patients in 16 cases, price mark-up schemes in 13 and price cuts in 11. Sales volumes increased moderately in all countries except Greece and Portugal, which experienced slight declines after 2009. Sales values decreased in both groups of countries, but fell more in less stable countries.

**Conclusion:**

Less economically stable countries implemented more pharmaceutical policy changes during the recession than economically stable countries. Unexpectedly, pharmaceutical sales volumes increased in almost all countries, whereas sales values declined, especially in less stable countries.

## Résumé

**Objectif:**

Identifier les changements de politique pharmaceutique au cours de la récession économique dans huit pays européens et déterminer si les mesures politiques ont entraîné une baisse des ventes et des dépenses en matière de produits pharmaceutiques.

**Méthodes:**

Les informations relatives aux changements de la politique pharmaceutique entre 2008 et 2011 dans huit pays européens ont été obtenues à partir des publications et des bases de données sur la politique pharmaceutique. Les données sur le volume et la valeur des ventes trimestrielles des produits entre 2006 et 2011 pour les 10 classes thérapeutiques les plus vendues dans chaque pays ont été obtenues à partir d'une base de données d'études de marché sur les médicaments. Nous avons comparé ces indicateurs dans des pays économiquement stables: Autriche, Estonie et Finlande, par rapport aux indicateurs mesurés dans des pays moins stables: Grèce, Irlande, Portugal, Slovaquie et Espagne.

**Résultats:**

Les pays économiquement stables ont chacun mis en œuvre 2 à 7 changements de politique, tandis que les pays moins stables en ont mis en œuvre 10 à 22 chacun. Sur les 88 changements de politique identifiés, 33 ont eu lieu en 2010 et 40 en 2011. Ils impliquaient des changements dans les paiements restants à charge pour les patients dans 16 cas, des plans d'augmentation des prix dans 13 cas et des baisses de prix dans 11 cas. Les volumes des ventes ont augmenté modérément dans tous les pays à l'exception de la Grèce et du Portugal qui ont connu de légères baisses après 2009. Les valeurs des ventes ont diminué dans les deux groupes de pays, mais elles ont davantage chuté dans les pays moins stables.

**Conclusion:**

Les pays moins stables économiquement ont mis en œuvre plus de changements de politique pharmaceutique au cours de la récession que les pays économiquement stables. De manière inattendue, les volumes des ventes de médicaments ont augmenté dans presque tous les pays, alors que les valeurs des ventes ont baissé, en particulier dans les pays moins stables.

## Resumen

**Objetivo:**

Identificar los cambios en las políticas farmacéuticas en ocho países europeos durante la recesión económica y determinar si las medidas de política dieron lugar a una reducción en las ventas y a un gasto menor en productos farmacéuticos.

**Métodos:**

La información sobre los cambios en las políticas farmacéuticas entre 2008 y 2011 en ocho países europeos se obtuvo a partir de publicaciones y bases de datos sobre políticas farmacéuticas. Los datos sobre el volumen y valor de las ventas trimestrales de productos entre 2006 y 2011 en las 10 clases terapéuticas más vendidas en cada país se obtuvieron de una base de datos de investigación del mercado farmacéutico. Comparamos estos indicadores en los países económicamente estables (Austria, Estonia y Finlandia) con los de los países menos estables (Grecia, Irlanda, Portugal, Eslovaquia y España).

**Resultados:**

Cada uno de los países económicamente estables implementó entre dos y siete cambios de políticas, mientras que los países menos estables implementaron entre 10 y 22 cada uno. De los 88 cambios de políticas identificados, 33 tuvieron lugar en el año 2010 y 40, en el año 2011. Implicaron cambios en los pagos directos por atención sanitaria para pacientes en 16 casos, programas de aumento de precios en 13 casos y recortes de precios en 11. Los volúmenes de ventas aumentaron moderadamente en todos los países excepto en Grecia y Portugal, que experimentaron descensos ligeros a partir de 2009. El valor de las ventas disminuyó en ambos grupos de países, si bien fue mayor en los países menos estables.

**Conclusión:**

Los países económicamente menos estables implementaron más cambios en las políticas farmacéuticas durante la recesión que los países económicamente estables. Sorprendentemente, los volúmenes de ventas de productos farmacéuticos aumentaron en casi todos los países, mientras que el valor de las ventas disminuyó, especialmente en los países menos estables.

## ملخص

**الغرض:**

تحديد التغيرات في سياسة المستحضرات الصيدلانية أثناء الكساد الاقتصادي في ثمانية بلدان أوروبية، وتحديد ما إذا كانت تدابير السياسة قد أدت إلى انخفاض مبيعات المستحضرات الصيدلانية وانخفاض الإنفاق عليها.

**الطريقة:**

تم الحصول على معلومات عن التغيرات في سياسة المستحضرات الصيدلانية بين عامي 2008 و2011 في ثمانية بلدان أوروبية من المنشورات وقواعد بيانات سياسة المستحضرات الصيدلانية. وتم الحصول على بيانات بشأن حجم وقيمة المبيعات ربع السنوية للمنتجات بين عامي 2006 و2011 في الفئات العلاجية العشر الأعلى مبيعاً في كل بلد من قاعدة بيانات لأبحاث سوق المستحضرات الصيدلانية. وقمنا بمقارنة هذه المؤشرات في البلدان المستقرة اقتصادياً وهي النمسا وإستونيا وفنلندا بالمؤشرات في البلدان الأقل استقراراً وهي اليونان وأيرلندا والبرتغال وسلوفاكيا وإسبانيا.

**النتائج:**

نفذت البلدان المستقرة اقتصادياً من اثنين إلى سبعة تغيرات في سياسات كل منها، بينما نفذت البلدان الأقل استقراراً من 10 إلى 22 تغيراً في سياسات كل منها. وحدث 33 تغيراً في السياسات في عام 2010 و40 تغيراً في السياسات في عام 2011 من أصل 88 تغيراً في السياسات التي تم تحديدها. وشمل ذلك تغيير المدفوعات التي يتحملها المرضى من جيوبهم الخاصة في 16 حالة ونظم هامش ربح الأسعار في 13 حالة وخفض الأسعار في 11 حالة. وازدادت أحجام المبيعات على نحو متوسط في جميع البلدان باستثناء اليونان والبرتغال اللذين شهدا انخفاضات طفيفة بعد عام 2009. وانخفضت أحجام المبيعات في مجموعتي البلدان، غير أنها انخفضت على نحو أكبر في البلدان الأقل استقراراً.

**الاستنتاج:**

نفذت البلدان الأقل استقراراً من الناحية الاقتصادية المزيد من التغيرات على سياسة المستحضرات الصيدلانية أثناء الكساد عن البلدان المستقرة اقتصادياً. وازدادت أحجام مبيعات المستحضرات الصيدلانية، على نحو غير متوقع، في جميع البلدان تقريباً، بينما انخفضت أحجام المبيعات، لا سيما في البلدان الأقل استقراراً.

## 摘要

**目的:**

识别八个欧洲国家在经济衰退中的医药政策变化，并判别政策措施是否导致药品销售量和支出的减少。

**方法:**

从出版物和药品政策数据库获取八个欧洲国家2008年和2011年之间医药政策变化信息。从医药市场研究数据库中获得每个国家2006年到2011年之间10个销量最高治疗类产品季度销售数量和价值的数据。我们将奥地利、爱沙尼亚和芬兰等经济稳定的国家与希腊、爱尔兰、葡萄牙、斯洛伐克和西班牙等经济不稳定国家的这些指标进行比较。

**结果:**

在经济稳定的国家中，每个国家实施二至七项政策变化，而在经济不稳定的国家中，每个国家实施10至22项政策变化。在识别的88项政策变化中，33项发生在2010年，40项发生在2011年。其中16个案例涉及改变患者自付政策，13个案例牵涉到改变标价制度，11个案例与改变降价方案相关。除了希腊和葡萄牙在2009年出现轻微的下降外，所有国家的销售量都适度增长。两组国家的销售价值都有所降低，但经济不稳定国家下降更多。

**结论:**

较之经济稳定的国家，经济不太稳定国家在经济衰退时期实施更多医药政策变化。出人意料的是，几乎所有国家药品销量都有增加，而销售价值则下降，在经济不稳定国家尤其如此。

## Резюме

**Цель:**

Идентифицировать изменения фармацевтической политики в период экономического спада в восьми европейских странах и определить, привели ли политические меры к снижению продаж фармацевтических препаратов и расходов на них.

**Методы:**

Информация об изменениях в фармацевтической политике за период 2008-2011 гг. в восьми европейских странах была получена из публикаций и баз данных фармацевтической политики. Данные об объемах и суммах квартальных продаж продукции за 2006-2011 гг. для десяти самых продаваемых терапевтических классов в каждой стране были получены из базы данных исследований фармацевтического рынка. Были сопоставлены показатели экономически стабильных стран (Австрия, Эстония и Финляндия) с подобными показателями менее стабильных стран (Греция, Ирландия, Португалия, Словакия и Испания).

**Результаты:**

В каждой из экономически стабильных стран было реализовано от 2 до 7 изменений в политике, в то время как в каждой из менее стабильных странах – от 10 до 22 изменений. Из 88 выявленных изменений в фармацевтической политике 33 произошли в 2010 году и 40 – в 2011 году. Они включали: изменения неофициальных расходов для пациентов в 16 случаях, схемы повышения цен в 13 случаях и снижение цен в 11 случаях. Объемы продаж умеренно выросли во всех странах, за исключением Греции и Португалии, где имелось незначительное снижение после 2009 года. Продажная стоимость снизилась в обеих группах стран, причем в более значительной степени в менее стабильных странах.

**Вывод:**

Экономически менее стабильные страны реализовали больше изменений фармацевтической политики в период экономического спада, чем экономически стабильные страны. Неожиданным результатом явилось то, что объемы продаж фармацевтической продукции увеличились практически во всех странах, тогда как продажная стоимость снизилась, особенно в менее стабильных странах.

## Introduction

European public authorities struggle to maintain a high level of access to health care while restraining increases in expenditure associated with an ageing population and higher demand.^1^^–^^4^ The recent global economic recession has put additional pressure on public budgets.^5^^,^^6^

In 2008, Europe was affected by the financial crisis. As the recession in Europe continued, the effect was felt especially in southern European countries and Ireland in 2010 and 2011. Soon the problem of financial debt for individual European countries developed into a crisis in the Eurozone, which then became a high priority for the European Central Bank and the European Parliament. All countries were urged to implement cost-saving measures that affected public financing for health care.^7^

Recession, which is defined as two successive quarters of negative growth in gross domestic product (GDP), can have a detrimental effect on the health of the population because economic downturns are strongly associated with a decline in health-care utilization and a deterioration in health outcomes.^8^ For example, suicides and homicides increased among working-age men and women when unemployment rose rapidly during past recessions in Europe.^9^ In the current recession, the number of uninsured non-elderly Americans increased by 5.6 million between 2007 and 2009^10^ and over a quarter of Americans reported reduced routine use of medical care.^11^ Over the same period, insurance policy deductibles and copayments for visits to physicians and for prescription medicines increased, leading to a greater cost burden for patients.^12^^–^^14^ Similar effects were seen in Greece. Studying the health effects of the economic crisis in the country it was found that patients had less access to care and preventive services and, consequently, faced higher risks of infection with sexually transmitted diseases.^15^ The World Health Organization examined the influence of the recession on expenditure on, and the sales and prices of, medicines between 2007 and 2009 in 84 countries. It found that the economic recession had mixed effects and that the largest declines in medicine sales occurred in high-income countries and in Europe, particularly in the Baltic states.^16^

It has been shown that countries that were seriously affected by the crisis, such as the Baltic countries, Greece, Portugal and Spain, abruptly implemented several pharmaceutical policy measures between 2010 and 2011. This included price cuts, changes in reimbursement rates and the imposition of value-added tax on medicines.^17^ In other European countries, such as Italy, in which cost-containment measures were already in place when the crisis began, the implementation of planned policy changes was accelerated.^18^

Because different countries were affected differently by the recession and attempted to overcome budgetary constraints in different ways, we decided to analyse systematically how European pharmaceutical policies were affected by the recession by comparing changes in pharmaceutical pricing and reimbursement policies between economically stable and economically less stable countries. In addition, we investigated changes in the sale of pharmaceuticals in major therapeutic classes before and after the recession in these two types of countries. We expected that some of the cost-containment policies, such as those affecting out-of-pocket payments, would shift the financial burden of medicines onto patients and hypothesized that pharmaceutical sales would decline during this period, especially in less economically stable countries.

## Methods

### Data sources

For this longitudinal study, we used data from two sources to derive information on pharmaceutical policies: (i) the Pharmaceutical Pricing and Reimbursement Information Network (Austrian Health Institute, Vienna, Austria), which collects information from experts in national pharmaceutical pricing and from authorities responsible for reimbursement – the latter provide regular pharmaceutical policy updates; and (ii) the PharmaQuery database (IMS Health, Philadelphia, United States of America), which contains data on pharmaceutical policies. In addition, we included information on policy changes reported in the published literature. We grouped policy changes into 6-month implementation periods from January 2008 until December 2011 and we categorized policy as relating to one of three main areas: (i) pricing; (ii) reimbursement; and (iii) generic drugs. Table 1 defines the policy measures in these three areas.

**Table 1:** National policy measures influencing pharmaceutical sales^19^

Policy measure	Definition
**Pricing**	
Price cut	A cost-containment measure whereby the set price of a medicine is reduced by the authorities.
External price referencing	External price referencing is the practice whereby the price of a medicine in one or several other countries is used to derive a benchmark or reference price for the purpose of setting or negotiating the medicine’s price in a given country.
	Policy changes in external price referencing include the introduction or abolition of this pricing policy and altering the methodology (e.g. changing the basket of reference countries or the way of calculating the benchmark price).
Distribution remuneration (i.e. mark-ups, margins and fees for service)	Distribution remuneration is the payment of a health-care provider, whether an individual or an organization, for the services provided. In the distribution of pharmaceuticals, wholesalers and pharmacies are remunerated using mark-ups or regressive margin schemes or, for pharmacies alone, by paying a “fee for service”. With mark-ups, a defined linear or percentage amount is added to the cost of a good to ensure a profit at the wholesale or retail level or both. With regressive margin schemes, the margin is expressed as a percentage of the selling price.
	Policy changes in distribution remuneration include adjusting the mark-ups or margins used for wholesalers or pharmacies or changing the type of distribution remuneration for a defined actor. Changes may also be made to the types of medicines (e.g. reimbursable medicines or prescription-only medicines) to which distribution remuneration applies.
VAT on medicine	VAT is a sales tax on products that is collected in stages. It is a wide-ranging tax that is usually designed to cover most or all goods and services, including medicines.
	Policy changes in VAT include the introduction or abolition of VAT on medicines and altering the VAT rate on medicines.
Extraordinary price review	Price reviews involve reviewing the process by which the set price of a medicine was established. Reviews may or may not be performed in combination with reimbursement reviews. Reviews can be performed systematically (e.g. once a year) for all reimbursed medicines or for a group of medicines (e.g. for a specific indication) or at any time.
**Reimbursement**	
Reference price system	With a reference price system, which is also referred to as internal or therapeutic reference pricing, the third party payer determines a reference price for the reimbursement of medicines with a particular active ingredient or in a given therapeutic class. If the price of the medicine exceeds the reference price, the health-care consumer must pay the difference between the fixed reimbursed amount (i.e. the reference price) and the actual pharmacy retail price in addition to any copayments (e.g. prescription costs and percentage copayment rates).
	Policy changes in the reference price system include the introduction or abolition of a reference price system and changing the methodology by which clusters of medicines are established for determining a reference price (e.g. by grouping identical or similar medicines).
Out-of-pocket payments	Out-of-pocket payments are payments made by health-care consumers that are not reimbursed by a third-party payer. They include cost-sharing, fixed or percentage copayments and informal payments to health-care providers.
Delisting	Delisting is the exclusion of a medicine from a reimbursement list (e.g. a positive list), which often results in exclusion from reimbursement.
**Generic drugs**	
INN prescribing	With INN prescribing, prescribers (e.g. physicians) are required to prescribe medicines using the INN for the pharmaceutical (i.e. the name of the active ingredient) instead of a brand name.
	Policy changes in INN prescribing include its introduction or abolition, changing the way INN prescribing is organized (e.g. by imposing or eliminating financial incentives) and changing from indicative to obligatory INN prescribing.
Generic substitution	Generic substitution is the practice of substituting a medicine, whether marketed under a trade name or generic name (i.e. a branded or unbranded drug), by a less expensive medicine (e.g. a branded or unbranded generic drug), which often contains the same active ingredients. Generic substitution may be encouraged (i.e. indicative generic substitution) or required (i.e. mandatory generic substitution).
	Policy changes in generic substitution include its introduction or abolition, changing the way generic substitution is organized (i.e. imposing or eliminating financial incentives) and moving from indicative to obligatory generic substitution.
Public campaigns	Policies, regulations, measures and initiatives promoting the use of generic drugs or licensed, off-patent medicines are typically undertaken by government authorities. Policy on generic drugs may be targeted at prescribers, pharmacists, patients or consumers, or other stakeholders.

INN: international nonproprietary name; VAT: value-added tax.

Quarterly pharmaceutical sales data for the period January 2006 to December 2011 were obtained from the IMS MIDAS (Multinational Integrated Data Analysis System) Quantum pharmaceutical market research service (IMS Health, Philadelphia, USA). Data were expressed in standard units for the volume of sales and in constant United States dollars (US$) for the value of sales. A standard unit, as defined by IMS Health, is the smallest dose of a product – it may be one tablet or capsule for oral preparations, one teaspoon (i.e. 5 mL) for a syrup or one ampoule or vial for an injectable product. The value of sales was derived from the price deemed most accurate for the relevant country and was expressed in constant US$, which were calculated by converting the local currency into United States dollars at a constant exchange rate. In most countries, the price used was the ex-factory price; in Estonia, Finland, Greece and Ireland, ex-factory prices were derived from wholesale prices. Average standard conversion factors, which were determined with the co-operation of the pharmaceutical industry for each country, were applied to estimate prices at various points along the distribution chain. The price calculations did not take into account any discounts between manufacturers, wholesalers and payers and were not adjusted for inflation.

Our study considered only prescription medicines, whether on or off patent, that were available in the retail market for the 10 highest-selling therapeutic classes. We identified the 10 highest-selling classes by ranking therapeutic classes according to their sales volume in each country. Together the combined sales volume of products in these 10 classes accounted for at least 50% of the total sales volume of all medicines in each of the eight countries from 2008 to 2011 (Table 2). Data were aggregated by therapeutic class for each country. We had no data on individual drugs.

**Table 2:** Ten highest-selling^a^ therapeutic drug classes in eight European countries,^b^ 2008–2011

Third-level code of the ATC classification^20^	Therapeutic class
A10C, A10H, A10J, A10K, A10L, A10M, A10N, A10S and A10X	Antidiabetes products
A02B	Antiulcer products
B01C	Platelet aggregation inhibitors
C10A, C10C and C11A	Lipid regulators
C09A and C09B	ACE inhibitors, either as single agents or in combination with other antihypertensives
M01A and M02A	Antirheumatics
N02A	Non-narcotic analgesics
N06A	Antidepressants
R01A6, R01B and R06A	Antiallergy drugs: systemic and nasal preparations and topical products
R03A, R03B, R03C, R03D, R03E, R03F, R03G, R03H, R03I, R03J and R03X	Respiratory agents

ACE: angiotensin-converting enzyme; ATC: Anatomical Therapeutic Chemical.

^a^ Together the products in these classes accounted for at least 50% of sales by volume in each country under investigation.

^b^ Austria, Estonia, Finland, Greece, Ireland, Portugal, Slovakia and Spain.

### Country groups

We considered eight European countries in which the majority of the population was covered by a social security system or national health service: Austria, Estonia, Finland, Greece, Ireland, Portugal, Slovakia and Spain. We selected these countries because they represented a variety of geographical regions and levels of economic wealth and stability and had been affected by the recession to different degrees. We classified them as either economically less stable or economically stable using categories defined by the Organisation for Economic Co-operation and Development (OECD) for the level of fiscal consolidation in 2012. Fiscal consolidation was judged according to whether the country had adopted either concrete policies aimed at stabilizing general government gross debt or a long-term target for the debt-to-GDP ratio of 60%. There were four categories of country: (i) those that had adopted a programme proposed by the International Monetary Fund, the European Union and the European Commission (e.g. Greece, Ireland and Portugal); (ii) those that were under clear market pressure (e.g. Belgium, Hungary, Italy, Slovakia and Spain); (iii) those that had a substantial deficit or debt but which were under less market pressure (e.g. Austria, Denmark, Finland, France and Germany); and (iv) those that had no or only a marginal need for consolidation (e.g. Norway, Sweden and Switzerland).^21^ In this study, we regarded economically less stable countries as those belonging to the first two categories (i.e. Greece, Ireland, Portugal, Slovakia and Spain) and economically stable countries as those belonging to the third and fourth categories (i.e. Austria, Estonia and Finland).

### Data analysis

First, we described and analysed the number of policy measures implemented per year, per country group and per policy category. Next, we determined the volume and value of the sales of drugs in each therapeutic class between 2006 and 2011 in each country and, then, we calculated the combined volume and value of the sales of drugs for all 10 therapeutic classes for each country. Since our findings for individual therapeutic classes and for all therapeutic classes combined were similar, we present only the results for all therapeutic classes combined.

For this analysis, we divided the volume and value of sales by the size of the country’s population to control for population growth; annual population figures were obtained from the OECD.^22^

We derived the annual and average growth rates over the study period using both the volume and value of pharmaceutical sales per capita:

$$\text{AGR}=\left[\frac{S_{\text{y}}}{S_{\text{y}-1}}-1\right]\times 100$$ (1)

$$\text{AAGR}=\frac{\sum \text{AGR}}{n}$$ (2)

where AGR is the annual growth rate, *S*_y_ is the per capital sales in a year, *S*_y–1_ is the per capital sales in the previous year, AAGR is the average annual growth rate and *n* is the number of years.

To compare changes in the volume and value of sales, we calculated the difference between the annual growth rate in the value of pharmaceutical sales and the annual growth rate in the volume of sales for each country.

## Results

### Changes in policy

Table 3, Table 4 and Table 5 (available at: http://www.who.int/bulletin/volumes/92/9/13-129114) summarize the 88 policy changes we identified in pricing, reimbursement and generic drugs, respectively. Economically stable countries implemented 7 or fewer policy changes each between 2008 and 2011; the lowest number was 2 in Finland (Table 6). Less economically stable countries implemented between 10 and 22 changes each; the highest number was 22 in Portugal. The greatest number of policy adjustments occurred in 2010 (33) and 2011 (40) and the most frequently used policy measures involved changes in out-of-pocket payments by patients (16), changes in regulations controlling the mark-up of prices (13) and price reductions (11). Some countries implemented several pricing measures. For example, Spain enacted four price cuts between 2008 and 2011. Most changes concerned reimbursable medicines and built on existing policies; only a few changes involved newly implemented policies, such as the introduction of internal reference pricing in Finland.^17^

**Table 3:** Policy changes on pharmaceutical pricing in eight European countries,^a^ 2008–2011

Policy measure	Implementation date of change in policy							
	1st half of 2008	2nd half of 2008	1st half of 2009	2nd half of 2009	1st half of 2010	2nd half of 2010	1st half of 2011	2nd half of 2011
Price cut	None	Portugal: 30% price cut for generics	Portugal: 5–12% price cut for generics	None	Greece: emergency price cuts up to a maximum of 27% for all reimbursed medicines (except orphan drugs); off-patent medicines cut to 90% of original cost. Ireland: 40% price cut for off-patent medicines. Spain: First price cut of up to 30% for generics; second price cut of 7.5% for health-care products, including original medicines, imposed in the form of a discount shared by all actors in the supply chain; in addition, a 4% price cut for orphan drugs and a 20% price cut for incontinence products	Portugal: 7.5% price cut for biological medicines and HIV products. Spain: price increase of 10–20% for amoxicillin-containing medicines to prevent their withdrawal from the market	Ireland: price cuts	Greece: 35% price cut for on-patent medicines before patent expiry and 15% price cut for generics. Spain: gradual price decreases
External price referencing	None	None	None	Greece: calculation method changed. Slovakia: reference countries changed	Portugal: calculation method changed. Spain: calculation method changed	None	Greece: calculation method changed. Slovakia: calculation method changed	Greece: calculation method changed. Portugal: reference countries changed
Distribution remuneration	None	None	None	Ireland: wholesale remuneration changed	Portugal: wholesale remuneration changed (i.e. the linear margin for nonreimbursable medicines was increased); pharmacy remuneration changed (i.e. the linear margin for nonreimbursable medicines was increased). Spain: pharmacy remuneration changed (i.e. part of the pharmacy remuneration for expensive medicines was increased)	None	Estonia: wholesale remuneration changed. Greece: wholesale remuneration decreased; supply chain discounts abolished. Ireland: wholesale remuneration for high-cost medicines changed (the High-Tech Scheme); wholesale remuneration for the general scheme for low-income patients decreased. Spain: wholesale remuneration changed; pharmacy remuneration changed	Greece: pharmacy remuneration changed. Portugal: wholesale and pharmacy remuneration changed in February 2012 from linear to regressive remuneration, which was effectively a decrease
VAT on medicine	None	None	Austria: VAT decreased from 20% to 10%. Estonia: VAT increased from 5% to 9%	None	Ireland: VAT increased to 21% for non-oral preparations. Portugal: VAT increased from 5% to 6%	None	Greece: VAT decreased from 11% to 6.5%. Spain: VAT increased from 8% to 10% for health-care products	None
Extraordinary price review	None	Ireland: review of reimbursed medicines	None	None	Portugal: review of selected active substances. Spain: price review, taking price cuts into account	Greece: review of new price lists, taking price cuts into account. Ireland: review of brands and parallel imports.^b^ Slovakia: review of reimbursed medicines	None	Greece: review resulted in a new price list that included an average 10.2% price reduction

HIV: human immunodeficiency virus; VAT: value-added tax.

^a^ Austria, Estonia, Finland, Greece, Ireland, Portugal, Slovakia and Spain.

^b^ A parallel import is the import of a patented or trademarked product from a country where it is already marketed, often at a lower cost.

**Table 4:** Policy changes on pharmaceuticals reimbursement in eight European countries,^a^ 2008–2011

Policy measure	Implementation date of change in policy							
	1st half of 2008	2nd half of 2008	1st half of 2009	2nd half of 2009	1st half of 2010	2nd half of 2010	1st half of 2011	2nd half of 2011
Reference price system	None	None	Finland: introduction of internal reference pricing	None	Portugal: price–volume agreement specified	Estonia: calculation method changed. Slovakia: reference price system changed to take Greek price cuts into account	Portugal: price calculation method changed and new generic groups introduced. Slovakia: new clusters of medicines established. Spain: price calculation changed to use lowest daily treatment costs as a basis	Greece: pricing changed to be at or below the reference price
Out-of-pocket payments	Austria: prescription fee increased	None	Austria: prescription fee increased. Portugal: reimbursement rate increased from 95% to 100% for generics for low-income pensioners; reimbursement rate increased from 37% to 69% for infertility drugs	None	Austria: prescription fee increased. Portugal: abolition of out-of-pocket payments for organ, tissue and stem cell transplant procedures	Ireland: introduction of € 0.50 payment per prescription medicine. Portugal: reimbursement rates changed for all medicines, including antipsychotics. Spain: underprivileged patients in Madrid given free access to products for seven rare diseases	Austria: prescription fee increased. Estonia: elimination of copayment limit. Slovakia: cost-sharing agreements implemented. Spain: copayment linked to patient’s income	Portugal: faster reimbursement reviews. Slovakia: limits imposed on certain reimbursement categories; reimbursement list to be published more frequently
Delisting	None	None	Finland: Seroquel delisted	None	None	None	Greece: introduction of a negative list of excluded products that included contraceptives and lifestyle medicines. Portugal: delisting of 16 branded nonprescription medicines, including paracetamol, oral omeprazole, contraceptives and antihistamines. Spain: delisting of selected medicines	Greece: 49 medicines were delisted after a price review

^a^ Austria, Estonia, Finland, Greece, Ireland, Portugal, Slovakia and Spain.

**Table 5:** Policy changes on generic drugs in eight European countries,^a^ 2008–2011

Policy measure	Implementation date of change in policy							
	1st half of 2008	2nd half of 2008	1st half of 2009	2nd half of 2009	1st half of 2010	2nd half of 2010	1st half of 2011	2nd half of 2011
INN prescribing	None	None	None	None	None	Estonia: change from optional to compulsory generic prescribing	Slovakia: optional generic prescribing introduced	Spain: optional generic prescribing introduced. Portugal: compulsory generic prescribing specified
Generic substitution	None	None	None	None	None	None	None	None
Public campaigns and other generic policies	Austria: generics information campaign	None	None	None	Estonia: generic campaign	Estonia: e-prescribing introduced. Ireland: rebates for generics abolished. Portugal: campaign to promote rational medicines use; dispensing of unit doses rather than sealed packages; prices to be displayed on packaging. Slovakia: establishment of health technology assessment institute. Spain: national generics campaign	Spain: unit dose dispensing introduced for four substances	Portugal: e-prescribing introduced and opening hours of pharmacies changed. Portugal: entry of 25 active generic substances into the market to be expedited subject to resolution of patent disputes

INN: international nonproprietary name.

^a^ Austria, Estonia, Finland, Greece, Ireland, Portugal, Slovakia and Spain.

**Table 6:** Policy measures influencing pharmaceutical sales in eight European countries, 2008–2011

Policy measure	No. of measures implemented between 2008 and 2011^a^									Total
	Economically stable countries^b^				Economically less stable countries^b^					
	Austria	Estonia	Finland		Greece	Ireland	Portugal	Slovakia	Spain	
**Pricing**										
Price cuts	0	0	0		2	2	3	0	4	11
External price referencing	0	0	0		3	0	2	2	1	8
Distribution remuneration	0	1	0		3	3	3	0	3	13
VAT on medicines	1	1	0		1	1	1	0	1	6
Extraordinary price review	0	0	0		2	2	1	1	1	7
**Reimbursement**										
Internal reference pricing	0	1	1		1	0	2	2	1	8
Out-of-pocket payments	4	1	0		0	1	5	3	2	16
Delisting	0	0	1		2	0	1	0	1	5
**Generics**										
INN prescribing	0	1	0		0	0	1	1	1	4
Generic substitution	0	0	0		0	0	0	0	0	0
Public campaigns and other generic policies	1	2	0		0	1	3	1	2	10
**Total**	**6**	**7**	**2**		**14**	**10**	**22**	**10**	**17**	**88**

INN: international nonproprietary name; VAT: value-added tax.

^a^ The number of measures implemented in each year was: 4 in 2008; 11 in 2009; 33 in 2010; and 40 in 2011.

^b^ The three economically stable countries implemented 15 measures during 2008–2011 compared with 73 in the five economically less stable countries.

### Changes in sales

**Fig. 1:**
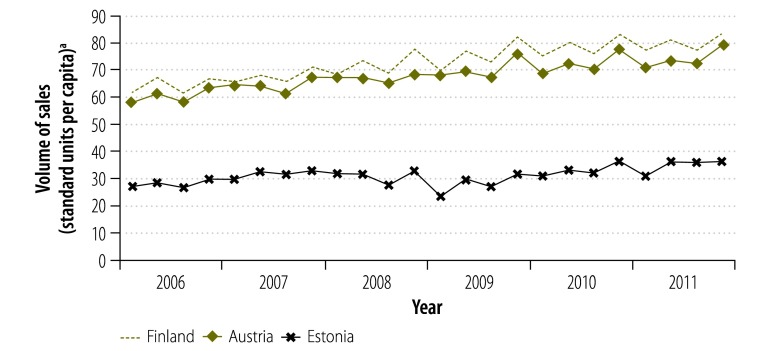
Volume of pharmaceutical sales, quarterly, in three economically stable European countries, 2006–2011

^a^ The volume of pharmaceutical sales is for the 10 highest-selling therapeutic classes in each country.

**Fig. 2:**
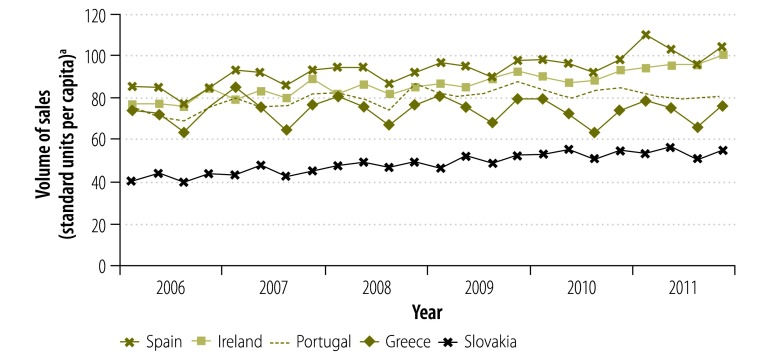
Volume of pharmaceutical sales, quarterly, in five economically less stable European countries, 2006–2011

^a^ The volume of pharmaceutical sales is for the 10 highest-selling therapeutic classes in each country.

**Fig. 3:**
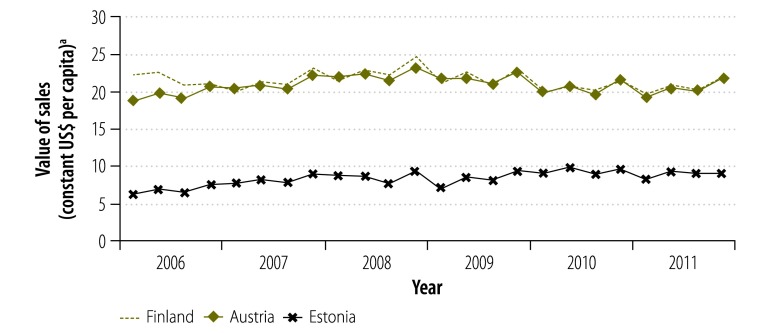
Value of pharmaceutical sales, quarterly, in three economically stable European countries, 2006–2011

**Fig. 4:**
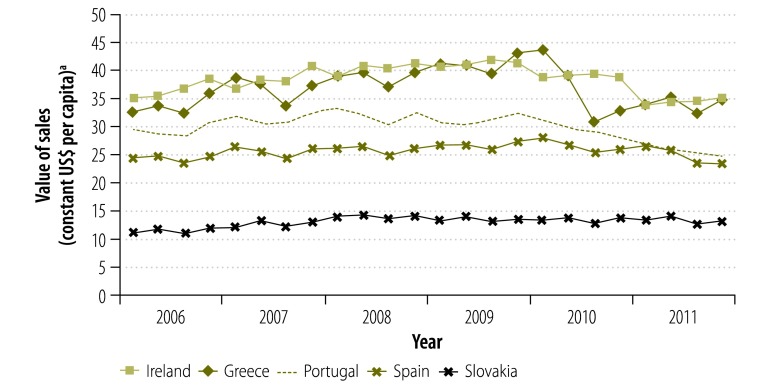
Value of pharmaceutical sales, quarterly, in five economically less stable European countries, 2006–2011

The small increase in the volume of pharmaceutical sales in all countries between 2006 and 2011 is shown in Fig. 1, Fig. 2, Fig. 3, Fig. 4 and Table 7: the average annual per capita growth in sales volume ranged from 0.8% in Greece and 1.0% in Portugal to 3.7% in Ireland, 4.0% in Slovakia and 4.6% in Estonia. However, annual growth rates were much more variable: from 2006 to 2007 the growth rate was over 3.7% for all countries, with Estonia having the highest rate at 12.2%. Between 2007 and 2009, growth remained fairly stable in Austria and Finland but there was a sharp decline in Estonia: the annual growth rate was −0.5% from 2007 to 2008 and −9.0% from 2008 to 2009. The growth rate declined in all economically less stable countries but more gradually. After the steep year-on-year decline in Estonia in 2009, the volume of sales grew 17.1% from 2009 to 2010. In contrast, the volume continued to decline in economically less stable countries: for example, from 2009 to 2010, there was a decline of −4.1% in Greece and −0.5% in Portugal. From 2010 to 2011, two of the less economically stable countries experienced a high growth in sales volume (5.5% in Spain and 7.8% in Ireland), while the growth rate was between 1.0% and 3.1% in most other less economically stable countries. The exception was Portugal, which experienced a decline of −3.7%.

US$: United States dollars.

^a^ The value of pharmaceutical sales is for the 10 highest-selling therapeutic classes in each country.

US$: United States dollars.

^a^ The value of pharmaceutical sales is for the 10 highest-selling therapeutic classes in each country.

**Table 7:** Per capita growth in pharmaceutical sales for the 10 highest-selling therapeutic classes in eight European countries, by volume and value, 2006–2011

Country	Per capita annual sales growth (%)					
	2006–2007	2007–2008	2008–2009	2009–2010	2010–2011	Average for 2006–2011
**Volume of sales^a^**						
Economically stable countries						
Austria	4.6	4.0	2.7	1.5	1.1	2.8
Estonia	12.2	−0.5	−9.0	17.1	3.1	4.6
Finland	3.7	3.8	2.3	3.4	1.0	2.8
Economically less stable countries						
Greece	5.6	0.3	0.7	−4.1	1.5	0.8
Ireland	4.1	1.4	4.3	0.8	7.8	3.7
Portugal	6.1	1.8	1.1	−0.5	−3.7	1.0
Slovakia	6.1	7.1	1.7	4.1	1.0	4.0
Spain	6.4	0.2	1.5	0.7	5.5	2.9
**Value of sales^b^**						
Economically stable countries						
Austria	7.3	6.3	2.2	0.4	1.5	3.5
Estonia	20.5	5.2	0.3	7.0	−3.2	6.0
Finland	3.1	6.3	−2.2	−2.6	0.7	1.1
Economically less stable countries						
Greece	13.3	7.0	6.8	−13.5	−2.4	2.2
Ireland	7.6	7.2	3.6	−1.9	−3.4	2.6
Portugal	5.4	2.0	−2.2	−4.6	−11.1	−2.1
Slovakia	12.0	14.6	0.6	0.5	−0.9	5.4
Spain	6.1	3.1	2.7	−0.4	−3.7	1.6

^a^ The volume of sales was measured in standard units per capita (IMS Health, unpublished data, 2012).

^b^ The value of sales was measured in constant United States dollars per capita (IMS Health, unpublished data, 2012).

The average annual per capita growth in the value of sales between 2006 and 2011 varied between −2.1% in Portugal and 6.0% in Estonia. After 2009, all countries except Austria experienced a decrease in the value of sales in at least one year. The largest annual declines were observed in Greece (−13.5% from 2009 to 2010) and Portugal (−11.1% from 2010 to 2011). Moreover, the value of sales declined from 2010 to 2011 in all economically less stable countries. 

**Fig. 5:**
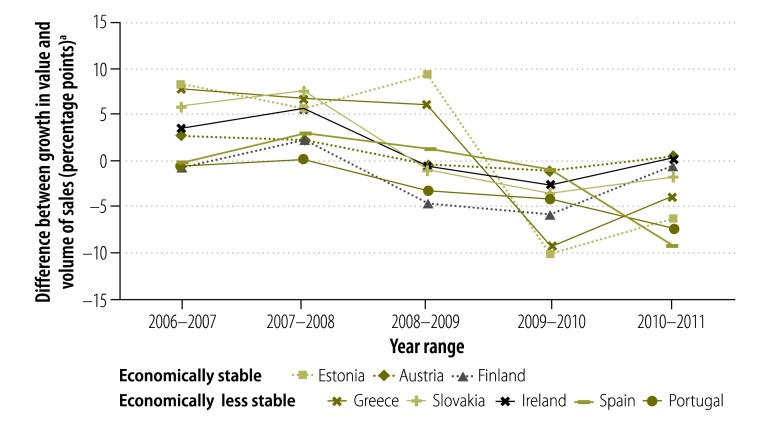
Difference between the annual growth in the value and volume of pharmaceutical sales,^a,b^ in eight European countries,^c^ 2006–2011

^a^ The graph illustrates the percentage point difference between the annual percentage growth in the value of pharmaceutical sales for the 10 highest-selling therapeutic classes and the annual percentage growth in the volume of sales. The volume of sales was measured in standard units per capita and the value of sales was measured in constant United States dollars (US$) per capita.

^b^ A negative value implies that the volume of sales increased more in a year than the value of sales, which indicates a decrease in the average price per unit.

^c^ Austria, Estonia, Finland, Greece, Ireland, Portugal, Slovakia and Spain.

Fig. 5 depicts the difference between the annual growth rate in the value of pharmaceutical sales and the annual growth rate in the volume of sales in each country between 2006 and 2011. In general, between 2006 and 2008, the annual value of pharmaceutical sales increased more than the annual volume of sales in both economically stable and less stable countries, which indicates that the average price per unit increased. From 2009 onwards, during the period when most policy changes were implemented, the growth in the annual value of sales was less than the growth in the annual volume, which indicates a decrease in average price per unit.

## Discussion

Although countries adjust their pharmaceutical policy frameworks continuously, a surge of policy changes seems to have taken place during the economic recession, particularly in 2010 and 2011. Unexpectedly, both economically stable and economically less stable countries experienced a slight increase in the consumption of pharmaceuticals in the 10 highest-selling therapeutic classes, as measured in standard units per capita. As expected, the annual growth in the per capita value of medicine sales decreased in economically less stable countries in 2010 and 2011.

Our study shows that economically stable countries implemented fewer policy measures between 2008 and 2011 than economically less stable countries. The most frequently implemented policy changes targeted out-of-pocket payments for patients. Previous studies have shown that increases in copayments, such as prescription fees, tend to lead to lower medicine utilization, especially in times of economic recession and increased unemployment.^23^^–^^30^ Policy measures such as the medicine price cuts (also applied in the form of discounts) that were implemented in Greece, Portugal and Spain could have had a negative effect on the availability of medicines if they caused pharmaceutical companies to withdraw their products from national reimbursement lists.^31^ Contrary to our expectations, however, we did not observe a major decline in the consumption of pharmaceuticals during the recession in the therapeutic categories studied as most countries continued to experience a moderate positive annual growth in sales volume. However, in line with media reports of drug shortages in Greece and Portugal, our data showed that the sales volumes of important medicines for chronic diseases, such as angiotensin-converting enzyme inhibitors and antidepressants, dropped drastically in these two countries in 2010.^31^ Hence, although the overall growth in sales volume was positive, the rate of growth appears to have fallen to below the prerecession level, which ranged from 5% to 12%.

In contrast, the rate of growth in the value of pharmaceutical sales declined, especially in economically less stable countries. This decrease may have been due partly to inflation: the average inflation rate in 2010 and 2011 generally ranged between 2.0% and 3.4%, although it was as low as −1.6% in 2010 in Greece and as high as 5.1% in 2011 in Estonia.^32^ Our analysis did not take inflation into account. The decrease may also have occurred because the pharmaceutical policies implemented in economically less stable countries had the desired effect of lowering public spending while maintaining access to medicines at a relatively stable level. For example, utilization could have shifted to less expensive or generic medicines. Nevertheless, even if sales volumes were maintained at lower prices, since several policy measures probably increased out-of-pocket payments for patients, the financial burden on patients may have increased.

The case of Estonia needs to be discussed separately. After a decade of rapid growth before the recession, during which public sector expenditure grew 6.5 times,^21^ Estonia experienced a major decline in GDP in 2009. Public sector spending was cut by 6.6% – a reduction of 100 million euros compared with 2008 – and there was a 50 million euros reduction in health insurance expenditure.^33^ A previous study identified a large decline in the consumption of pharmaceuticals of −18% between 2008 and 2009,^16^ which was mirrored in our data. In response, Estonia implemented strict cost-saving measures with respect to medicines, reduced sick leave coverage and increased the workload of clinical staff without increasing their salaries.^16^^,^^33^ Our data show that, by 2010, the consumption of pharmaceuticals had returned to a level similar to that before the recession, which paralleled Estonia’s relatively quick recovery from the recession overall.^21^

Early in the recession, countries not only implemented few policies changes overall but also implemented no policies that targeted consumption by specific patient groups or in specific therapeutic areas. Recent studies show that fewer policy changes were implemented in 2012 and 2013 than during the recession and that there was a trend towards policies that targeted high-cost medicines.^34^ Several countries have explored alternative policies for sharing the financial risk of selected, new, high-cost medicines, such as value-based pricing models or risk-sharing agreements.^35^^–^^38^ The effect of these new approaches still needs to be determined.

Our study had several limitations. We did not take into account the differences in pharmaceutical policy frameworks that existed between all countries before the economic recession or between regions within some countries (e.g. Italy or Spain). Moreover, it was not always clear whether a country implemented a policy as a short-term reaction to recession-related budgetary constraints or whether the policy was part of a planned long-term change to the system. For instance, in Finland, the implementation of internal reference pricing in 2009 had been planned long before the recession.^39^ Major policy changes, such as the introduction of reference price systems, may take several years to implement since many stakeholders are involved.^40^ However, most of the policy changes related to the recession involved adjusting existing policies and could be implemented relatively quickly. Although these policies might, as desired, contain costs over the short-term, they could have substantial long-term effects on the use and affordability of medicines and could have negative consequences for health.^41^^–^^44^ We focused our analysis on the sale of products that accounted for the majority of pharmaceutical sales by volume. It is possible that policy changes had a differential influence on the sale of less frequently used products, including those used by patients with rare diseases. However, at least one price cut in Greece exempted orphan drugs for rare diseases.

Another limitation is that, since data on the value of sales are expressed in constant US$ and disregard discounts and rebates, they do not reflect actual spending by third-party payers. In addition, an individual country’s data might include different products within each therapeutic class. Moreover, medicines sales may also be influenced by other market variables, such as patent expiration. During the study period, patents expired on several highly used products, including diabetes medications, antiulcerants, platelet aggregation inhibitors, lipid regulators, angiotensin-converting enzyme inhibitors and antidepressants. The price reductions accompanying patent expiries may have combined with policies promoting generic prescribing to reduce the value of sales while limiting the decline in sales volume. Finally, the rapid implementation and the different timing of policies in different countries meant that we were unable to attribute the observed change in pharmaceutical sales to any single policy or set of policies or to make statistical comparisons of responses to policy between countries.

We suggest that future research focuses on the effect of policy changes in only a few countries by exploring the relationship between changes in medicine utilization and health outcomes. Moreover, since some of our findings were not in line with our expectations, we recommend that studies of the effect of new pharmaceutical policies should monitor access to medicines and look for potential barriers to access.

In conclusion, the ways in which countries responded to the recession differed greatly, with less economically stable countries implementing a larger number of policies that affected the pharmaceutical sector than economically stable countries. Our evidence shows that, despite numerous policy changes and contrary to our expectations, overall consumption of pharmaceuticals in the 10 highest-selling therapeutic classes continued to increase in most countries; there was no clear difference between economically stable and less stable countries. The observation that the value of sales declined while the volume was maintained may indicate that pharmaceutical purchasing became more efficient. However, since many policies were designed to shift the financial burden to patients, future research should investigate the effect of changes in pharmaceutical policy, expenditure and utilization on equitable access to medicines, on the affordability of essential medicines for households, on the appropriate use of medicines and on health outcomes.
